# *In vivo* functional analysis of a nuclear restorer PPR protein

**DOI:** 10.1186/s12870-014-0313-4

**Published:** 2014-11-18

**Authors:** Xike Qin, Richard Warguchuk, Nadège Arnal, Lydiane Gaborieau, Hakim Mireau, Gregory G Brown

**Affiliations:** Department of Biology, McGill University, 1205 Doctor Penfield Ave., Montreal, QC H3A 1B1 Canada; INRA, UMR1318, Institut Jean-Pierre Bourgin, RD10, F-78000 Versailles, France; AgroParisTech, Institut Jean-Pierre Bourgin, RD10, F-78000 Versailles, France; Current address: Lady Davis Institute for Medical research, 3999 Cote Ste-Catherine Rd., Montreal, QC H3T 1E2 Canada; Current address: Deparment of Pharmacology and Therapeutics, McGill University, 3655 Promenade Sir-William-Osler, Montréal, QC H3G 1Y6 Canada; Current address: INRA, Centre National de Ressources Génomiques Végétales, Castanet Tolosan, France

**Keywords:** PPR protein, Targeted mutagenesis, Cytoplasmic male sterility, Nuclear restorer gene, Mitochondria, Structure-function relationship

## Abstract

**Background:**

Nuclear restorers of cytoplasmic male fertility (CMS) act to suppress the male sterile phenotype by down-regulating the expression of novel CMS-specifying mitochondrial genes. One such restorer gene is *Rfo*, which restores fertility to the radish Ogura or *ogu* CMS. *Rfo*, like most characterized restorers, encodes a pentatricopeptide repeat (PPR) protein, a family of eukaryotic proteins characterized by tandem repeats of a 35 amino acid motif. While over 400 PPR genes are found in characterized plant genomes and the importance of this gene family in organelle gene expression is widely recognized, few detailed *in vivo* assessments of primary structure-function relationships in this protein family have been conducted.

**Results:**

In contrast to earlier studies, which identified 16 or 17 PPR domains in the Rfo protein, we now find, using a more recently developed predictive tool, that Rfo has 18 repeat domains with the additional domain N-terminal to the others. Comparison of transcript sequences from pooled *rfo/rfo* plants with pooled *Rfo/Rfo* plants of a mapping population led to the identification of a non-restoring *rfo* allele with a 12 bp deletion in the fourth domain. Introduction into *ogu* CMS plants of a genetic construct in which this deletion had been introduced into *Rfo* led to a partial loss in the capacity to produce viable pollen, as assessed by vital staining, pollen germination and the capacity for seed production following pollination of CMS plants. The degree of viable pollen production among different transgenic plants roughly correlated with the copy number of the introduced gene and with the reduction of the levels of the ORF138 CMS-associated protein. All other constructs tested, including one in which only the C-terminal PPR repeat was deleted and another in which this repeat was replaced by the corresponding domain of the related, non-restoring gene, *PPR-A*, failed to result in any measure of fertility restoration.

**Conclusions:**

The identification of the additional PPR domain in Rfo indicates that the protein, apart from its N-terminal mitochondrial targeting presequence, consists almost entirely of PPR repeats. The newly identified *rfo* allele carries the same 4 amino acid deletion as that found in the neighboring, related, non-restoring PPR gene, *PPR-A*. Introduction of this four amino acid deletion into a central domain the Rfo protein, however, only partially reduces its restoration capacity, even though this alteration might be expected to alter the spacing between the adjoining repeats. All other tested alterations, generated by deleting specific PPR repeats or exchanging repeats with corresponding domains of *PPR-A*, led to a complete loss of restorer function. Overall we demonstrate that introduction of targeted alterations of *Rfo* into *ogu* CMS plants provides a sensitive *in vivo* readout for analysis of the relationship between primary structure and biological function in this important family of plant proteins.

**Electronic supplementary material:**

The online version of this article (doi:10.1186/s12870-014-0313-4) contains supplementary material, which is available to authorized users.

## Background

Cytoplasmic male sterility (CMS) is a widespread trait in flowering plants specified by novel, often chimeric genes in the maternally inherited mitochondrial genome [1]. The trait can be suppressed by nuclear restorer of fertility (Rf) genes that act to specifically down-regulate the expression of corresponding novel, CMS-specifying, mitochondrial genes. The phenomenon of CMS and nuclear fertility restoration is of commercial interest because it can be used for the production of higher yielding hybrid crop varieties. From an evolutionary standpoint, maternally inherited male sterility may spread in a population of hermaphroditic plants due to different selective factors acting on nuclear and cytoplasmic genomes [2]. The consequent increase in frequency of females in the population will reduce pollen production [3] and create selective pressure for the appearance of a new nuclear restorer gene. In this sense, the phenomenon of CMS and fertility restoration can be viewed as a conflict between the nuclear and cytoplasmic genomes analogous to the “gene for gene” concept for conflict between genomes of host plants and their pathogens, an “intragenomic arms race” that has apparently been occurring throughout much of angiosperm evolutionary history [3-6].

Most characterized nuclear restorer genes have been found to encode pentatricopeptide repeat (PPR) proteins. PPR proteins are characterized by tandem degenerate repeats of a 35 amino acid motif, and most are thought to function as sequence-specific RNA binding proteins that modulate mitochondrial and chloroplast gene expression through post-transcriptional processes including editing, splicing and nuclease cleavage [7]. Most sequenced eukaryotic genomes possess only a few PPR encoding genes, but in land plants this gene family is greatly expanded and in angiosperm species it can encompass between 400 and 600 members, many of which are plant-specific variant forms with PPR-related repeats that are both longer and shorter than the degenerate core 35 amino sequence [8]. In general nuclear, restorer genes have been found to specify prototype P-type PPR proteins - those with tandem repeats consisting exclusively of the 35 amino acid core motif [7]. Phylogenetic analysis of the plant P-type PPR family indicates that restorer proteins are members of a distinct clade of “Rf-like” or *RFL* proteins restricted to flowering plants [3]. The *RFL* proteins within a given species appear to be under positive selective pressures, consistent with the intragenomic arms race hypothesis [3,6].

Like the related tetratricopeptide repeat (TPR) and PUF-domain proteins, the PPR domain, as initially proposed by Small and Peeters [9] is configured as two anti-parallel alpha-helices, with successive PPR domains forming an extended superhelix surrounding a central groove that functions in RNA binding [10,11]. Recently, combinatorial code models have been proposed that correlate key amino acid residues within a given repeat with its RNA binding properties [12,13]. Such models can explain the RNA binding specificity for some PPR proteins with known RNA binding sites and are consistent with some aspects of the solved structure of a PPR protein with its bound RNA ligand [11]. These models are not predictive of the binding sites for all PPR proteins and there have been few experimental investigations on the relationship between the structure of PPR proteins and their biological functions *in vivo* [14].

The radish (*Raphanus sativus*) nuclear restorer gene *Rfo* [15,16] is structurally identical to the radish *Rfk1* restorer [17] of the related radish Kosena CMS and restores fertility to *Brassica napus* plants carrying the *Ogu*-INRA form of the Ogura or *ogu* cytoplasmic male sterility [18,19]. The Rfo protein has been shown to be localized to mitochondria and to bind the target mRNA of the *ogu* CMS-associated gene, *orf138* [20]. Transgenic introduction of a single copy of *Rfo* into *Ogu*-INRA CMS plants is sufficient to completely restore male fertility [16,20]. *Rfo* is flanked in the radish genome by two other potential PPR-encoding sequences, g24 and g27 [16] corresponding to *PPR-A* and *PPR-C,* respectively, of Desloire et al. [15]^a^. The premise that *PPR-C* encoded a protein comparable in size to Rfo and PPR-A was based on the existence of a predicted intron that could not be verified by transcript analysis and it is therefore likely that this sequence is a pseudogene. Both the *PPR-A* and *PPR-C* genes are highly similar to *Rfo*, but each has a deletion that removes 4 amino acids from one of the PPR domains and both display a complete inability to restore fertility to *Ogu*-INRA CMS plants. Although it is likely that *PPR-C* is a pseudogene, *PPR-A* is expressed, though at a lower level than *Rfo* [20].

We have used the transgenic restoration of male fertility as a convenient means of assessing the biological function of variant forms of *Rfo*, and thereby probing primary structure-function relationships in this important category of plant proteins. We show here that significant primary structural perturbation of the Rfo protein completely eliminates its capacity to restore male sterility. Importantly, we have found that introduction into the Rfo protein of the four amino acid deletion found in PPR-A and PPR-C and a non-restoring radish allele of *Rfo* leads to a partial loss of restoration function that correlates with a partial loss of the capacity to suppress expression of the *ogu* CMS-associated protein ORF138. The degree of male-fertility varies in such plants in a manner dependent upon the copy number of the introduced transgene. The study indicates that transgenic introduction of *Rfo* or its variants into *Ogu*-INRA CMS *B. napus* plants provides a sensitive means of assessing PPR protein function that should facilitate dissection of primary structure-function relationships for this group of proteins.

## Results

### Rfo and PPR-A each contain 18 PPR motifs

The amino acid sequences of the Rfo proteins predicted in several different publications [15,16,20] are identical and identical to that for the protein predicted to be encoded by *Rfk1*, the nuclear restorer for a radish CMS system related to Ogura termed Kosena or *kos* [17]. Two groups [16,17], however, identified 16 PPR domains in the protein, while the third [15] reported an additional PPR domain located at the C-terminal side of the other domains. To clarify the number and location of PPR domains present in Rfo and the related PPR-A protein sequence, these sequences were analyzed by TPRpred [21], a resource that has been specifically designed to detect TPR, PPR and SEL1-like domains in protein sequences that may be missed by other methods.

**Figure 1:**
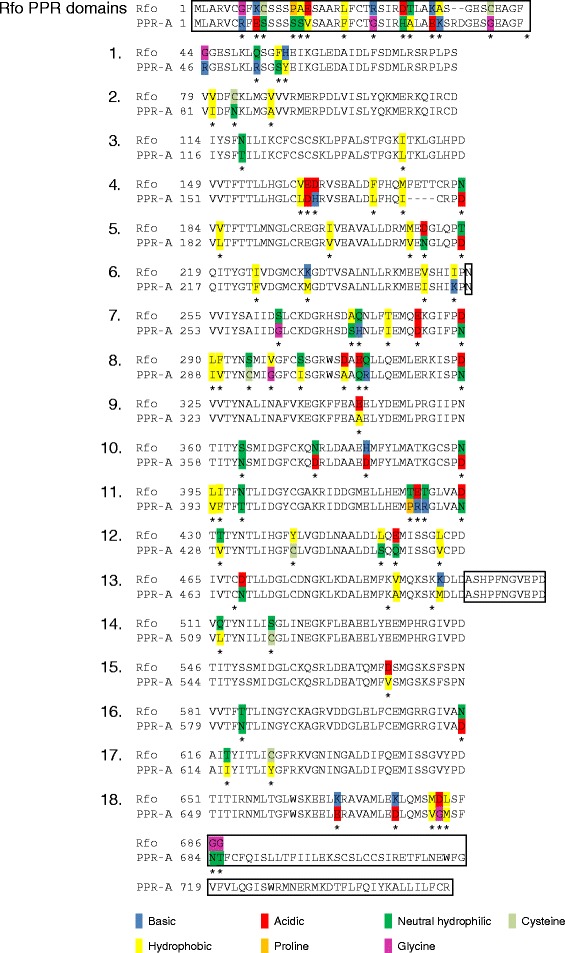
**PPR repeats in Rfo and PPR-A.** The eighteen PPR repeats detected by TPRpred in Rfo and the non-restoring adjacent protein PPR-A (designated gene 24 in [16]) are aligned, with each domain on a separate line. Amino acid differences between the two proteins indicated by asterisks (*) and highlighted according to the type of amino acid occurring in each protein. Portions of the proteins, including the N-terminal region containing the mitochondrial targeting sequence, that do not constitute a PPR domain are enclosed in boxes, with the exception of the portion of PPR-A corresponding to domain 4 of Rfo. The domains are presented on separate lines, numbered according to their position in the sequence and represented in the register proposed by Yin et al. [11] on the basis of the solved structure of a PPR protein bound to its RNA ligand. In this register the first amino acid of each domain corresponds to the second amino acid in the register employed in [3,12,15,20] and the last letter of the preceding domains detected by the Pfam resource [22] employed in [16] and [17].

As shown in Figure 1, TPRpred detects 18 PPR and 17 domains in the Rfo protein and PPR-A proteins, respectively (domain 4 of Rfo is not perceived as a PPR domain in PPR-A because of a 4 amino acid deletion [see below]). These domains include the 17 detected by Desloire and co-authors [13] as well as an additional domain closest to the N-terminus. TPRpred predicts domains that are in the same register or “frame” as the Pfam resource [22] employed by Koizuka et al. [17] and Brown et al. [16]. This register is shifted two amino acids towards the C-terminus from another widely employed PPR representation [3,12,15,20] corresponding to the initially proposed domain definition [9]. Thus residue 1 in the TPRpred/Pfam register corresponds to residue 3 in the latter register. More recently, a third representation of the PPR domain based on the solved 3-dimensional structure of a PPR protein was proposed [11]. In this representation the domains begin at residue 2 of Desloire et al. [15] and it is this register that is employed in Figure 1.

PPR-A is missing four amino acids found in domain 4 of Rfo, and has an additional stretch of 68 amino acids at its C-terminus that are not recognized as containing a PPR domain. It is interesting to note that the amino acids that have sustained the highest levels of replacement between Rfo and PPR-A occur at positions 2, 5 and 35 in the PPR domain. These residues are the same as those found to be subject to the highest levels of positive selection selective pressure [3] and two [12] or three [11] have been proposed to play a role in the recognition of the RNA ligand in combinatorial code models.

### A non-restoring *rfo* allele possesses the same 12 bp deletion in domain 4 as PPR-A

The presence of the same 12 bp deletion in the nucleotide sequences encoding both PPR-A and PPR-C raised the question as to whether this same deletion might be present in non- restoring *rfo* radish alleles. We thus searched for *rfo* sequences in the F5 Asian radish mapping population employed to clone *Rfo* [16], reasoning that such sequences would be found in sterile progeny but not in homozygous fertile progeny. During fine mapping studies of the *Rfo* gene using Arabidopsis-derived genetic markers, we noticed that nearly all the Arabidopsis probes employed as RFLP markers detected multiple, independently segregating genetic loci in the mapping population. This is consistent with the occurrence of genome duplication events in the Brassiceae tribe, which includes the radish genus Raphanus, subsequent to its divergence from the lineage leading to Arabidopsis [23], and has since been confirmed by detailed molecular mapping studies [24,25]. It was therefore essential that the potential *rfo* allele be identified as one that was clearly anchored at the *rfo* locus.

**Figure 2:**
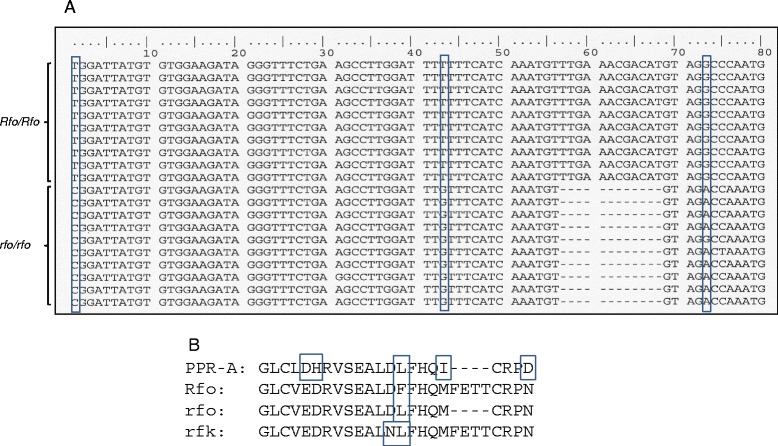
***rfo*** **alleles.** Panel **A** shows the nucleotide sequences of RT-PCR products obtained from pooled RNA samples of fertile plants of an F5 Asian radish mapping population [16] homozygous for the dominant *Rfo* restorer allele (*Rfo/Rfo*) or sterile plants (*rfo/rfo*) of the same population. Nucleotide differences between the two sets of sequences are enclosed in boxes. The amino acid sequences of various Rfo alleles and the corresponding region of PPR-A are illustrated in Panel **B**. rfk refers to the sterile allele of the *Rfk1* restorer of the related Kosena CMS system reported in [17]. The sequence of Rfk1 is identical to Rfo.

On the basis of this consideration, our approach to identifying the *rfo* allele in the sterile parent of this mapping population was to use the principle of bulked segregant analysis [26] to target the region spanning the PPR-A deletion. We specifically targeted the region spanning the PPR-A deletion by analyzing expressed sequences using *Rfo*-based primers to amplify transcript sequences from pooled RNA preparations made from individuals that were male-sterile or male-fertile and homozygous for *Rfo*-linked markers. As shown in Figure 2, one set of sequences was identified in this manner that had the same 12 nt deletion in comparison to *Rfo* as the PPR-A coding sequence. In comparison to *Rfo*, however, only three additional base substitutions were found in this sequence as opposed to 8 for *PPR-A*, and only one of these resulted in an amino acid substitution, as opposed to 5 for the *PPR-A* sequence. We therefore viewed the sequence as a likely allele of *Rfo* and not an ortholog of the *PPR-A* gene.

### A 12 bp deletion in *Rfo* domain 4 reduces but does not eliminate restorer function

**Figure 3:**
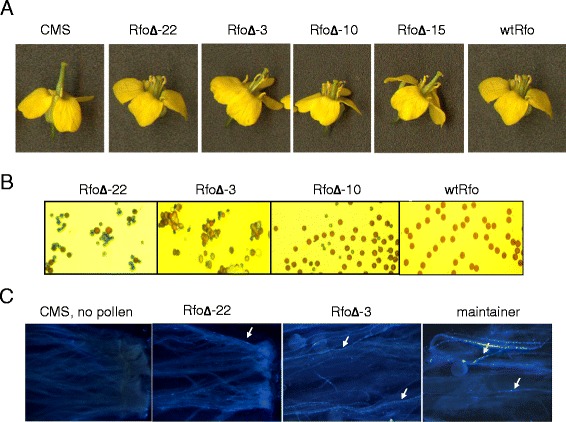
**Characterization of RfoΔ T0 generation phenotypes. (A)** From left to right, flowers from the non-transformed CMS recipient line, T0 RfoΔ transformants displaying progressively higher degrees of male fertility, and a completely male fertile plant transformed with the wtRfo construct. **(B)** Pollen grains from (left to right) progressively more sterile T0 RfoΔ transformants and a wtRfo transformant stained with the vital stain described by Alexander [29]; non-viable pollen stains green, viable pollen stains reddish brown. **(C)** Germination of pollen from a more sterile (plant 22) and a more fertile (plant 3) RfoΔ transformant and from the completely male fertile CMS maintainer line, cv “Westar”. The arrows point to brightly fluorescing extensively elongated tubes; for the more sterile plant RfoΔ-22 plant, only a few of the grains germinated to form long pollen tubes; more germination can be seen in the style pollinated by the more fertile RfoΔ-3 plant.

We chose to further investigate structure-function relationships in between Rfo and the related, non-restoring PPR proteins through directed mutagenesis experiments via consecutive PCR reactions [27,28]. Our initial construct, designated RfoΔ, was designed to determine the effect on fertility restoration of removing from Rfo the four amino acids that are missing from Rfo domain 4 in PPR-A and the non-restoring *rfo* allele. Approximately 30 T0 transgenic plants were recovered following its introduction into *ogu* CMS plants. *ogu* CMS flowers possess short filaments and anthers that remain closed and fail to shed pollen. In contrast, the recovered transgenic plants had longer stamens and anthers that opened and shed pollen (Figure 3A). Staining of the pollen of the RfoΔ transgenic plants, however, revealed that the proportion of viable pollen grains varied from plant to plant (Figure 3B). At one extreme were plants, here exemplified by plant 22, for which virtually all the grains stained green, characteristic of aborted, non-viable pollen [29]. Other plants, such as plants 3 and 10, showed a greater proportion of reddish-brown grains characteristic of viable pollen. Assessment of pollen viability in this manner correlated with its capacity to form pollen tubes in the styles of *ogu* CMS plants (Figure 3C). As expected, pollen from the more fertile partially restored plants also gave rise to more viable seeds following pollination of the *ogu* CMS line than did pollen from more sterile plants. Unexpectedly, however, we noticed that siliques formed following fertilization with pollen from the more fertile partially restored plants contained a relatively high proportion of smaller, aborted seeds that were incapable of germination (Table 1).

**Table 1:** **Seed production with pollen from RfoΔ plants**

	**Seeds per silique** ^**a**^	
**Cross**	**Mature**	**Aborted** ^**b**^
CMS x Maintainer	28	1.4
CMS x RfoΔ-3	1.2	8.6
CMS x RfoΔ-15	2.6	12.4
CMS x RfoΔ-16	7.6	14.2
CMS x RfoΔ-22	0.2	1.8

^a^The average numbers of each type of seed per silique based on 5 siliques from each plant. No significant differences were observed among the different siliques on a given plant.

^b^These seeds were markedly smaller than normal mature seed and were non-viable.

The degree of male sterility among the different transgenic individuals was found to correlate with the number of bands observed in Southern blot analysis using a probe consisting of the *Rfo* promoter and coding region (Additional file 1: Figure S1). Plants for which a high proportion of the pollen grains were non-viable showed only a single hybridizing band, suggesting the presence of only a single inserted copy, whereas plants releasing a higher proportion of viable pollen showed multiple hybridizing bands. Analysis of the pollen producing capacity of T1 plants generated by pollinating different T0 plants with the “Westar” maintainer line supported the view that the variation in pollen viability was a result of transgene copy number differences (Additional file 2: Table S1). Of the 20 plant 22 progeny, 8 had flowers that appeared phenotypically identical to *ogu* CMS plants, consistent with the presence of only one or two independently segregating copies of the transgene in the parent T0 plant. In contrast, all 20 of the progeny of the more fertile plants 3, 10 and 15 produced pollen, consistent with the presence of multiple copies of the gene in the parent T0 plants. Of the 5 progeny plants of a fertile wtRfo analyzed, four were completely fertile, and one was completely sterile, consistent with the presence of only a single copy of the transgene. We conclude from these experiments that the deletion of the 4 amino acids from PPR domain 4 of Rfo reduces but does not eliminate its function.

### Modifications of Rfo structure and expression that eliminate restorer function

**Figure 4:**
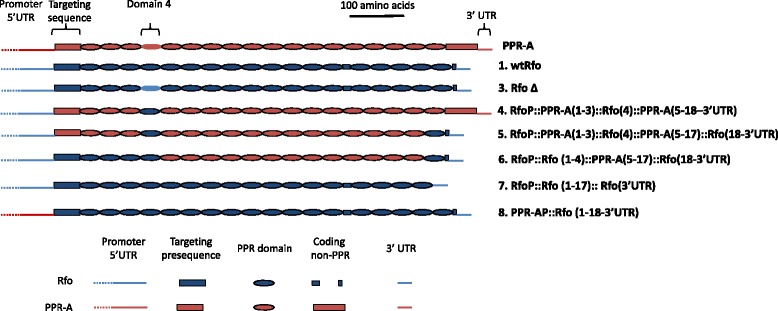
**Depiction of PPR-A, the base construct wtRfo and different derived genetic contructs.** PPR repeats are indicated by ovals, non-PPR domains by rectangles of equal height. Portions of the construct composed of PPR-A sequences are crimson, portions from Rfo sequences are blue. The domains with the 4 amino acid deletion in PPR-A and the RfoΔ construct lacks a black outline used to designate true PPR repeats. Non-protein coding regions are represented by lines. Construct numbers correspond to those of Additional file 6: Generation of genetic constructs. Construct 2 was omitted since it is a utility clone carrying only the *Rfo* promoter region with flanking Sal1 and Xho1 sites (Additional file 5: Figure S4).

Because of the high degree of variation observed among Rf-like PPR proteins [3,6] and the retention of partial Rfo function in the deletion allele, we reasoned that the functional constraints on Rfo and possibly other Rf-like PPR proteins might be relaxed. To further investigate this possibility, several other mutant forms which represented hybrid sequences, constructed by replacing domains from Rfo with their PPR-A counterparts, were introduced and expressed in *ogu* CMS plants. These constructs are depicted in Figure 4 and included sequences specifying a PPR-A protein in which the deletion carrying domain 4 was replaced with its Rfo counterpart (construct 4), a derivative of construct 4 in which the PPR-A domain 18, C-terminal extension and 3’UTR were replaced with the corresponding Rfo domains (construct 5) and a construct similar to construct 5, in which the PPR-A domains 1-4 were replaced by the corresponding Rfo domains (construct 6). Two additional constructs were investigated: in one, sequences encoding Rfo domain 18 and the two C-terminal amino acids (Figure 1) were removed (construct 7); in the other, the Rfo coding and 3’UTR sequences were expressed using the PPR-A promoter (construct 8).

For all these constructs, the flowers of transgenic plants recovered after transformation into *ogu*-CMS *B. napus* “Westar” appeared identical to those of the non-transformed CMS line and failed to produce pollen or set seed. Analysis of mitochondria from the progeny of crosses between a limited number of these plants and the Westar maintainer line with an anti-Rfo (PPR-B) antibody [20], an example of which is shown in Additional file 3: Figure S2, indicated that proteins encoded by the introduced sequences are expressed. While it is perhaps not surprising that constructs in which a significant number of Rfo domains were exchanged for their counterparts in PPR-A were not functional, the finding that removal of only the C-terminal PPR domain resulted in complete loss of function was unexpected. Also of interest was the finding that expression of an intact Rfo protein using the PPR-A promoter region also failed to restore any pollen production to CMS plants. Transgenic PPR-A protein is expressed from its native promoter at levels that are considerably lower than expression levels for transgenic Rfo using its promoter [20]. Thus, it seems likely that there needs to be at least a threshold level of Rfo expression for fertility restoration, and that expression with the PPR-A promoter is not sufficient to achieve this level.

### Expression of the CMS-associated ORF138 protein in partially restored plants

The *ogu* CMS is associated with expression of the novel mitochondrial gene *orf138* [30]. The protein encoded by this gene, ORF138, is strongly associated with the mitochondrial inner membrane, where it forms an oligomeric complex [31,32]. The level of this protein is reduced upon fertility restoration, which is consistent with its serving as the causative factor in *ogu* CMS [20,33,34]. The mechanism by which expression of this protein leads to CMS is unclear, however. From this standpoint, it was of interest to determine to what extent the levels of this protein correlated with the degree of male sterility expressed in the different transgenic lines generated upon transformation with the Rfo and RfoΔ constructs.

**Figure 5:**
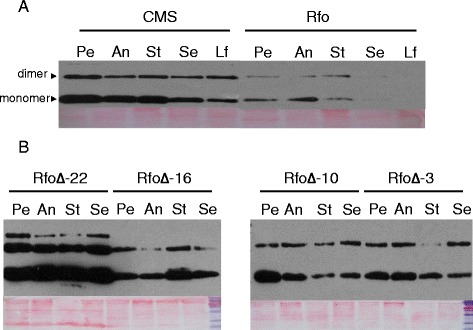
**ORF138 expression in CMS, fully restored and partially restored plants. (A)** Mitochondria were isolated from petals (Pe), anthers (An), styles (St), sepals (Se) and leaves (Le) of CMS and fully fertility restored *Rfo* transgenic plants. 5 μg of mitochondrial protein from each organ was subjected to immunoblot analysis with anti-ORF138 antisera. **(B)** Mitochondria isolated from different organs of T0 RfoΔ transgenic plants expressing different degrees of male sterility were subjected to SDS-PAGE and ORF138 was detected as in panel; RfoΔ-22 is the most male-sterile of the recovered plants. Panels below the immunoblots show the protein on the membranes as detected with Ponceau red.

The relative level of expression of ORF138 protein, as detected with anti-ORF138 antiserum in immunoblots of mitochondrial protein from different organs of CMS plants and plants expressing the wild type Rfo transgene, is shown in Figure 5A. In CMS plants, slightly higher levels of ORF138 expression are seen in the petals, anthers and style mitochondria than in the sepal and leaf mitochondria. ORF138 levels in all these tissues are markedly reduced in plants expressing the Rfo transgene, and the protein is barely detectable in mitochondria from sepals and leaves. Figure 5B shows a comparison of the ORF 138 levels in mitochondria from different RfoΔ plants expressing different degrees of male sterility. As expected, plant 22, which produced the lowest amount of viable pollen, showed the highest level of ORF 138 expression in all organs. Immunolocalization of ORF138 in the anthers of these plants indicated that it was expressed at highest levels in the tapetum as it is in CMS plants. Interestingly, among the plants with more intermediate levels of male sterility (plants 3, 10 and 16), there was little variation in the amount of ORF138 expression, although some differences in the degree of male sterility in these plants could be observed (Figure 4).

## Discussion

### PPR domains and recessive alleles of Rfo

Because of the differences in the numbers of Rfo PPR repeats recognized by the software applications employed by different research groups in previous analyses [15-17], we re-analyzed the Rfo and PPR-A sequences with TPRpred [21], an application developed after the initial publications on Rfo that is designed to detect more divergent TPR, PPR and SEL-1 solenoid repeat elements, and is capable of identifying more repeats, than applications previously used for this purpose, such as Pfam [22]. This approach allowed us to identify 18 PPR repeats in Rfo and 17 repeats in PPR-A, with the difference between the two proteins arising from the four amino acid deletion in the region of PPR-A that corresponds to domain 4 of Rfo. The 18 repeats included the domain closest to the C-terminus that was identified by Desloire et al. [15] as well as an additional repeat immediately following the N-terminal mitochondrial targeting sequence. Not surprisingly, the N- and C-terminal repeats had the highest of the reported P values of the TPRpred prediction, indicating they were the most diverged from the test sequences on which the computational Hidden Markov Model (HMM) is based. Interestingly, the C terminal repeat 18 previously reported [15] had a higher P value than the N-terminal repeat 1 and was not recognized as a PPR repeat in every TPRpred run of the Rfo sequence.

Previously, two different recessive alleles of the Rfo/Rfk1 protein have been reported. Koizuka et al [17] found that the sterile Asian radish line used to map the *Rfk1* gene [35] contained an expressed sequence with 11 base substitutions in the coding region, four of which caused amino acid substitutions in the encoded protein. Three of these occur in domain 4 and one in domain 3; the domain 3 substitution and one of the domain 4 substitutions occur at PPR residue 5, which the combinatorial code models predict to be involved in ligand recognition [12,13]. More recently a second *rfo* allele, in this case occurring in a European radish cultivar was described [36]. The *rfo* locus in this allele possessed only two PPR protein coding sequences, PPR-1 and PPR-2, which clustered more closely with PPR-A and PPR-C, respectively, than with Rfo (PPR-B). Both sequences were more highly diverged from *Rfo* than *rfk1*. We have now partially characterized a third *rfo* allele, in this case from an Asian radish variety, that encodes a protein with the four amino deletion also found in in PPR-A and PPR-C as well as one of the domain 4 amino acid replacements occurring in *rfk1*. This finding formed the initial basis for our further site directed mutagenesis study of the structural basis of Rfo function.

### Directed mutagenesis of *Rfo*

Expression of the RfoΔ construct, in which the Rfo sequence is missing the four amino acids of domain 4 that are deleted in *PPR-A* and the non-restoring *rfo* allele, led to only a partial loss of restoration capacity, as measured by several different criteria. The degree of fertility expressed in these transgenic plants also correlated well with the copy number of the introduced gene and with the degree to which expression of the CMS-associated polypeptide, ORF138 was reduced. It is noteworthy that none of the deleted amino acids correspond to key residues predicted to be involved in ligand recognition [11-13] and secondary structure prediction indicated the motif carrying the deletion would still assume the form of the beta (second) - helical segment of the PPR motif. The solved structure of a PPR protein bound to an RNA ligand shows that successive domains in the protein bind successive nucleotides in the RNA [11] and it has been suggested that the spacing of contiguous motifs in P-class PPR proteins may be important in limiting the length of contiguous nucleotides in the associated RNA sequence [12]. From this perspective, the retention of some biological activity in the deleted mutant form of the protein might be unexpected, since removal of these amino acids would likely affect the distance between adjacent domains and thereby possibly reduce its affinity for the *orf138* mRNA ligand and disrupt its capacity for fertility restoration. Although such disrupted spacing among repeats may reduce the affinity of Rfo for its RNA ligand, this does not evidently occur to the extent that biological function is completely abolished.

The degree of fertility of the different partially restored plants obtained with the RfoΔ construct also correlated with the number of viable seeds obtained following fertilization with the pollen produced by these plants. We also noted that a relatively high percentage of the seeds obtained using pollen from some of the plants were small and failed to germinate. This was unexpected, since the only known mode of action of Rfo is to suppress expression of ORF138 and ORF138 expression has no known effect on embryogenesis and seed development. Pollen development in *Ogu-I*NRA *B. napus* arrests at the uninucleate stage and the mitosis that gives rise to the generative cell does not occur [37]. While it is possible that the deletion allele has some additional effect on embryo development that is related to the aborted seed effect, it may also be that some RfoΔ pollen may develop past the uninucleate stage but experience a defect in the second pollen mitosis that leads to the formation of two generative cells. This would result in grains with only a single generative cell or with two defective generative cells. Fertilization events due to such pollen grains might then lead to defects in embryo and/or endosperm development. For example, pollen development in Arabidopsis *cdc2a* mutants is blocked at the second mitotic division; these grains are capable of fertilizing the egg cell but not the polar nuclei of the embryo sac, and the zygotes thus formed arrest early in embryogenesis [38].

In contrast to the phenotypes associated with the RfoΔ construct, the deletion of the C-terminal domain 18 resulted in an apparent complete loss of function, with no alteration in flower morphology or pollen production in comparison to the *ogu* CMS recipient line. Successive domains in PPR proteins bind successive residues in the RNA ligand [3,11-13] and conceivably this loss of function could reflect the importance of the binding of the terminal domain to forming a stable RNA-protein interaction.

When transgenic PPR-A is driven by its own promoter, it can be detected with antiserum that has similar sensitivity towards PPR-A and Rfo [20]. Its level of expression, however, is considerably lower than for corresponding constructs in which Rfo (PPR-B) is expressed off its promoter. The absence of any evidence of fertility restoration after introduction of the construct in which the *Rfo* promoter was replaced by the PPR-A promoter suggests that some critical level of expression of the protein must be obtained to achieve any level of evident suppression of the CMS phenotype. This might be the case if binding of Rfo to *orf138* mRNA were cooperative, although no evidence of cooperative binding has been observed in binding analyses of other PPR proteins, such as PPR10 [12]. A more likely explanation is that small decreases in the level of ORF138 expression have little or no effect on its disruption of pollen development and that more substantial suppression of its expression is required before significant amounts of pollen are released.

## Conclusions

We demonstrate here that directed mutagenesis of the *Rfo* nuclear restorer gene for the *Ogu*-INRA CMS of *Brassica napus* provides an effective and convenient means of assessing the function of individual domains and residues in a PPR protein. This approach takes advantage of the relatively facile, although cultivar dependent, capacity for Agrobacterium-mediated transformation of *B. napus* and the straightforward phenotype of fertility restoration. We show that the approach is sensitive enough to detect differences in the degree of fertility due, as least in part, to the copy number of a hypomorphic allele and that removal or replacement of domains with the corresponding domains of the related PPR gene, PPRA lead to an apparent complete loss of function. In addition, we show that an apparent threshold level of Rfo expression must be achieved for fertility restoration. It will be of interest to determine how the restoration activity of altered forms of the protein correlates with their affinity for the *orf138* RNA ligand.

## Methods

### Bioinformatic analysis of protein sequence

Protein sequences were analyzed using the on-line resources of the Bioinformatics Toolkit© of the Department of Protein Evolution, Max Planck Institute of Developmental Biology, Tübingen, Germany; these included TPRpred (http://toolkit.tuebingen.mpg.de/tprpred) for the prediction of PPR domains and Quick2D (http://toolkit.tuebingen.mpg.de/quick2_d) for secondary structural prediction.

### Identification of a non-restoring *rfo* allele

RNA was isolated and reverse transcriptase polymerase chain reaction (RT-PCR) analyses of the pooled RNAs was carried out using g26 primers P26-F3 and P26-R3 as described [16]. RT-PCR products were cloned using the TOPO TA Cloning® Kit (Invitrogen Life Technologies, San Diego, CA, USA). Using protocols provided by the supplier and sequenced by DNA LandMarks, Inc., St-Jean-sur-Richelieu, QC, Canada.

### Genetic constructs

The strategy used to generate amplicons for the various constructs, as exemplified by RfoΔ, is outlined in Additional file 4: Figure S3 and for cloning the amplicons in Additional file 5: Figure S4. We first generated a utility plasmid, RfoP, that would allow us to easily directionally insert coding-3’UTR sequence constructs immediately downstream of the *Rfo* promoter and provide a simple means of introducing these into the plant transformation vector. Constructs spanning coding and 3’UTR regions were then generated by sequential PCR reactions [27,28], introduced into the RfoP plasmid and excised and introduced into the plant transformation vector pRD400 [39]. Details of the amplification and cloning processes can be found in Additional file 6. Generation of genetic constructs.

### Plant transformation

Promoter/coding region clones were digested with Sal1 and EcoR1 and ligated into the corresponding sites on the plant transformation vector pRD400 [39]. The pRD400 clones were introduced into *Agrobacterium tumefaciens* strain GV3101 and transformed into INRA-*Ogu* cv. Westar *B. napus* as described [16].

### Pollen viability and pollen tube elongation

Pollen viability was estimated with Alexander stain [29]. Pollen grains were evaluated from newly opened flowers of wtRfo and RfoΔ transgenic plants and from the male-fertile maintainer line. Healthy viable pollen stains a deep-red to purple color whereas non-viable pollen stains green. Pollen from the male-fertile cv. Westar maintainer line and different RfoΔ transgenic plants was applied to the stigma of CMS plants; pollinated styles were removed 72 hours later and stained with aniline blue (0.1% in 0.1 M K_3_PO_4_) prior to microscopic inspection under ultraviolet light.

### Phenotypic analysis

CMS plants were fertilized with pollen from the maintainer line or from 5 RfoΔ transgenic plants. The number of seeds appearing in 5 randomly selected siliques of each cross were counted and classified as mature or aborted. To determine the additive effects of RfoΔ transgene copy number on male fertility, four RfoΔ plants were crossed with the maintainer line and a single wtRfo transgenic plant was self-crossed to serve as a control. Twenty plants of each cross were grown to maturity in a green house and the male fertility of individual progeny plants was evaluated.

### Southern blot analysis

Genomic DNA was extracted from leaf tissue of greenhouse grown plants as described [16]. 15ug samples from individual plants were digested with EcoR1 and eletrophoresed on a 1% agarose gel at 20 V for 20 hr, transferred to a charged nylon membrane (Amersham/GE Healthcare, Baie d’Urfe, QC). After 3 hr of blocking at 55°C with hybridization buffer plus blocking reagent (Amersham) and NaCl 0.5 M, a probe encompassing the wtRfo promoter and coding sequences was labeled with α-^32^PdCTP using the Prime-a-Gene Labeling system (Promega, Madison WI, USA). Hybridization was performed at 65°C overnight and the membrane was washed with 0.5% SSC and 0.5SDS% (W/V) 3 times for 5 minutes each at room temperature. The membrane was then washed twice for 30 minutes at 65°C and exposed to a Phosphor screen for 48 h at room temperature. The screen image was visualized using a PhosphorImager scanner (Amersham).

### Microscopy and immunohistochemical staining

Fixation, embedding and sectioning of 2-3 mm floral buds were performed according the Myerowitz laboratory protocol http://www.its.caltech.edu/~plantlab/protocols/insitu.pdf), with minor modifications. 8-10 μm cross sections were used for immunohistochemical staining by Vectastain Elite Kit (Vector Laboratories, Burlington, ON). In order to decrease background, endogenous peroxidase was blocked by Avidin/Biotin. ORF138 specific primary antibody was diluted in 1:500 immunohistochemical staining was performed according the manufacturer‘s instructions. Staining was monitored under a dissecting microscope after the addition of substrate, and 4 minutes was found to yield optimal signal intensity.

### Analysis of ORF138 and Rfo expression

Mitochondria from different plant tissues, isolated as described [40], were dissolved in SDS sample buffer (2% SDS, 2 mM mercaptoethanol, 4% glycerol, 40 mM TrisHCl pH 6.8, .01% bromphenol blue) and volume was adjusted give a final concentration of 5 μg protein per 20 μL buffer. After electrophoresis of 20 μL on a 10% SDS-PAGE gel, the proteins were transferred to nitrocellulose membranes and stained with 2% (w/v) Ponceau red. The membranes were blocked by an overnight incubation at room temperature with TBS-T (Tris-buffered saline containing 1.5% w/v BSA fraction V and 0.5% w/v Tween 80), incubated for 2 hr at room temperature with a 1:1000 dilution of anti-ORF 138 antibody, rinsed with TBS-T three times, and incubated for 4 hr in a 1:1000 dilution of anti-rabbit lgG-POD (Roche) in 5 ml TBS-T for 2 hr. After washing three times with TBS-T, the location of anti-rabbit IgG-POD was detected through the use of a BM Chemiluminescence Kit (Roche) and film detection. For detection of Rfo protein and its variants, T1 plants were raised to maturity, mitochondria were isolated from flower buds and proteins were detected with anti-Rfo protein was detected immunologically as described [20].

### Availability of supporting data

All of the supporting data is included as additional files. The sequence of Figure 2 corresponds to the reverse complement of nucleotides 3079 – 3158 of the *Rfo* gene sequence, GenBank AJ550021.2, http://www.ncbi.nlm.nih.gov/nuccore/AJ550021.2.

## Endnote

^a^We refer to the genes flanking the *Rfo* restorer using the more descriptive nomenclature of Desloire et al. [15] (i.e. *PPR-A* and *PPR-C*). We here use *Rfo* instead of *PPR-B* (Uyttewaal et al. [20]) to refer to the central PPR gene at the restorer locus, since it is evident that this sequence alone specifies restorer gene function.

## Additional files

Additional file 1: Figure S1. Southern blot analysis of individual Rfo and RfoΔ T0 transgenic plants. Additional file 2: Table S1. Segregation of male fertility sterility in the T1 progeny of wtRfo and RfoΔ transgenic plants. **Table S2.** Primers used for building genetic constructs. Additional file 3: Figure S2. Western analysis of transgenic Rfo and Construct 6 plants. Additional file 4: Figure S3. Strategy for generating site directed mutation, as exemplified by the assembly of the RfoΔ construct, in which a four amino acid deletion is introduced into PPR domain 4 of Rfo. Additional file 5: Figure S4. Strategy for cloning genetic constructs. Additional file 6:
**Generation of genetic constructs.**

## Abbreviations

CMS: Cytoplasmic male sterility
PPR: Pentatricopeptide repeat
