# Supplementary figures and images for: In vivo functional analysis of a nuclear restorer PPR protein
Source: BMC Plant Biol. 2014 Nov 18;14:313. doi: 10.1186/s12870-014-0313-4 (PMC4240901; doi:10.1186/s12870-014-0313-4)

A

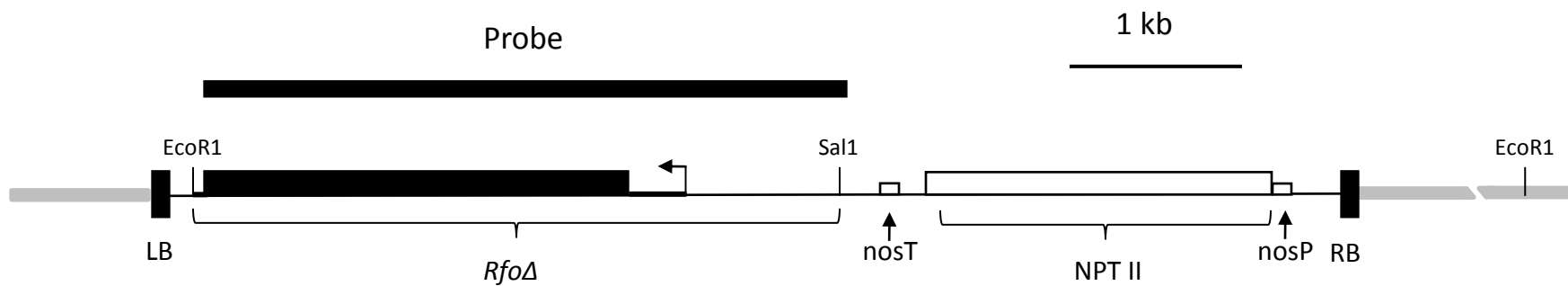

**B**

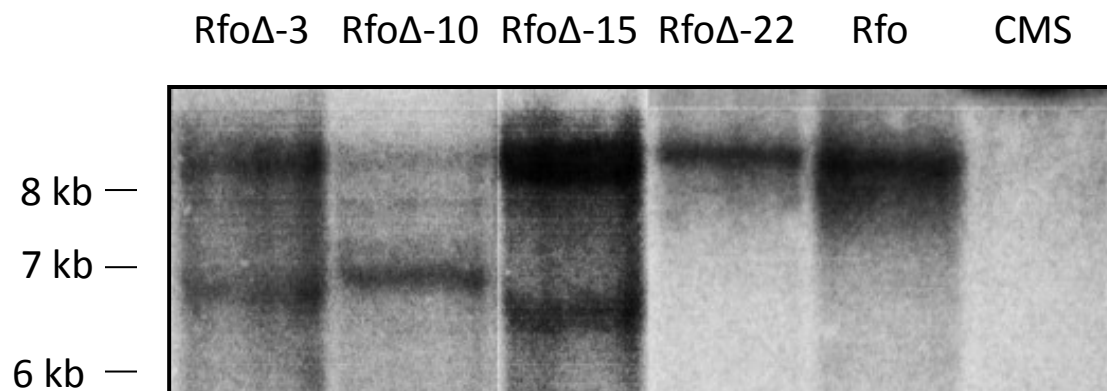

Supplement: Additional file 1: Figure S1. — Southern blot analysis of individual Rfo and RfoΔ T0 transgenic plants. [file 12870_2014_313_MOESM1_ESM.pdf]

CMS

Rfo

Construct 6

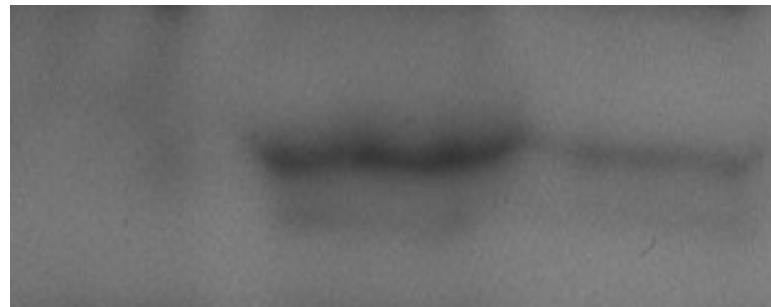

← 70 kD

Supplement: Additional file 3: Figure S2. — Western analysis of transgenic Rfo and Construct 6 plants. [file 12870_2014_313_MOESM3_ESM.pdf]

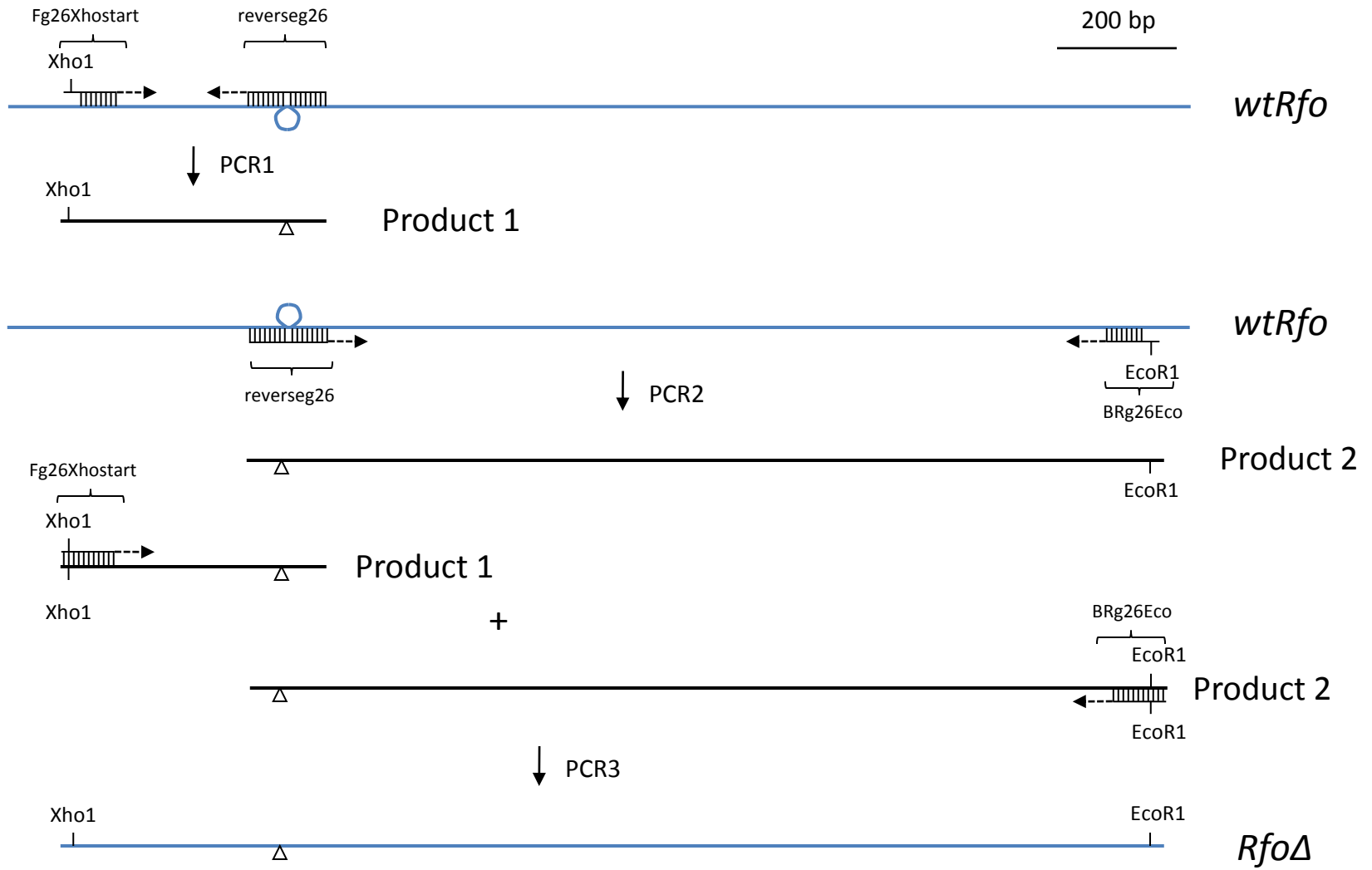

Supplement: Additional file 4: Figure S3. — Strategy for generating site directed mutation, as exemplified by the assembly of the RfoΔ construct, in which a four amino acid deletion is introduced into PPR domain 4 of Rfo. [file 12870_2014_313_MOESM4_ESM.pdf]

# Construct cloning strategy

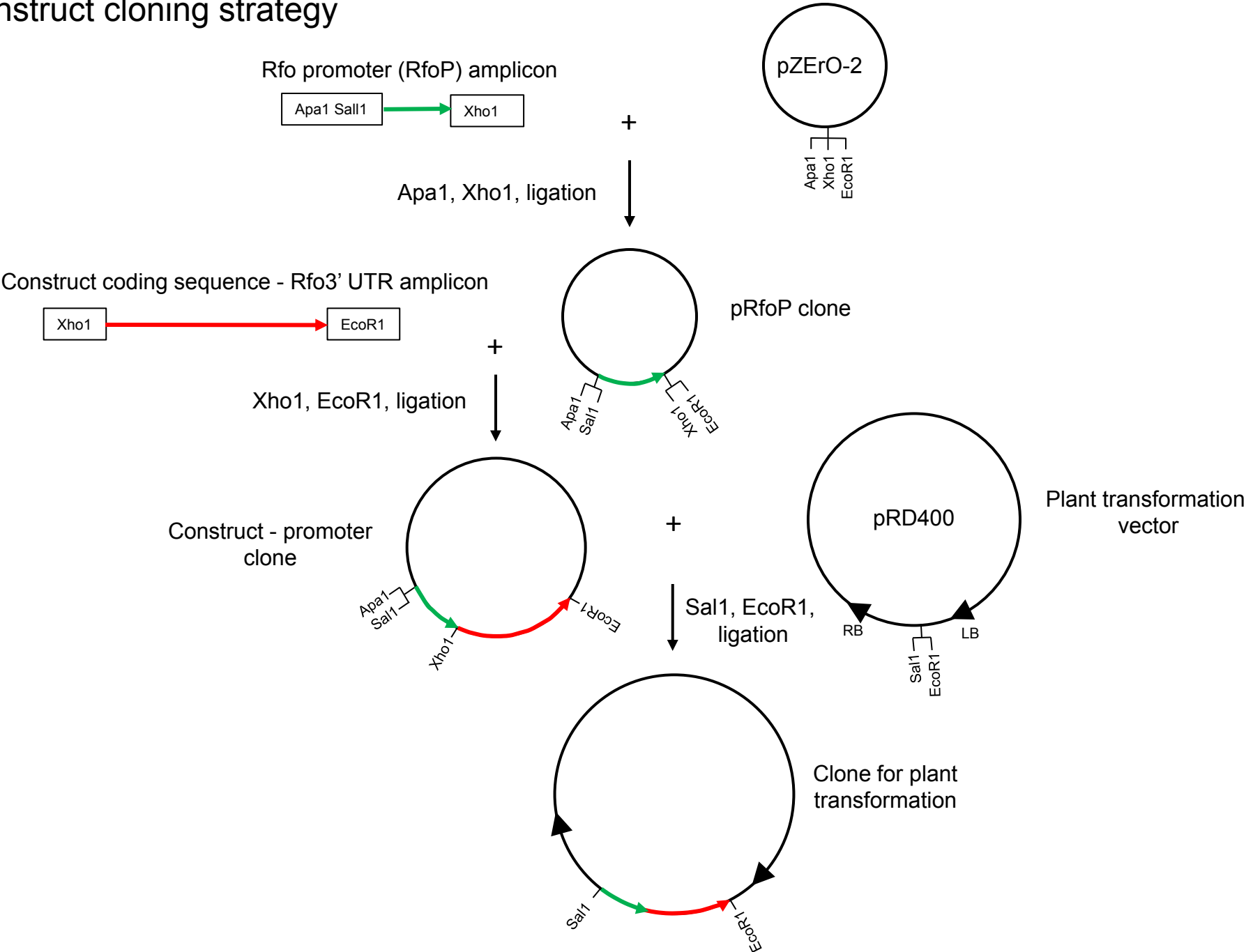

Supplement: Additional file 5: Figure S4. — Strategy for cloning genetic constructs. [file 12870_2014_313_MOESM5_ESM.pdf]
