# Supplementary material for: In vivo functional analysis of a nuclear restorer PPR protein
Source: BMC Plant Biol. 2014 Nov 18;14:313. doi: 10.1186/s12870-014-0313-4 (PMC4240901; doi:10.1186/s12870-014-0313-4)
Supplement: Additional file 2: Table S1. — Segregation of male fertility sterility in the T1 progeny of wtRfo and RfoΔ transgenic plants. Table S2. Primers used for building genetic constructs. [file 12870_2014_313_MOESM2_ESM.pdf]

SUPPLEMENTARY TABLES

Table S1. Segregation of male fertility sterility in the T1 progeny of wtRfo and RfoΔ transgenic plants

Cross	Pollen Production	
	Yes	No
RfoΔ-3 x Westar (maintainer)	20	0
RfoΔ-10 x Westar	20	0
RfoΔ-15 x Westar	20	0
RfoΔ-22 x Westar	12	8
wtRfo x wtRfo	5	1

Table S2. Primers used for building genetic constructs.

Primer designation	Sequence, 5’ to 3’	Construct number	Sequence: Location*
Sal1PF	**GAGAGTCGACGATTATTAAGATTTAATATAAATA**	1	Rfo: -1479 to -1456
BRg26Eco	**CTCTGAATTCTGGAAGGTGTGCCTCTTTAGTTC**	1,5,6,7,8	Rfo: +2468 to +2443
Pf	**GAGAGGGCCCGTCGACGATTATTAAGATTTAATATAAATA**	2	Rfo: -1479 to -1456
Pr	**CTCTCTCGAGTTTATTTTTGTTTCGCCTAAATTTTG**	2	Rfo: -1 to -26
Fg26Xhostart	**CTCTCTCGAGATGTTGGCTAGGGTTTGTGG**	3,6,7,8	Rfo: +1 to +20
Bforwardg26	**GCCTTGGATTTTTTTCATCAAATGTGTAGGCCCAATGTCGTAACCTTC**	3	Rfo: +502 to +526**<br>+539 to +561
reverseg26	**GAAGGTTACGACATTGGGCCTACACATTTGATGAAAAAAATCCAAGGC**	3	Rfo: +561 to +539**<br>+526 to +502
DFg24Xhostart	**CTCTCTCGAGATGTTGGCTAGGGTTTGCAGATTCG**	4, 5	PPR-A: +1 to +25
DRg24	**GAACGTTAGGACATCTGGTCTACATGTCGTTTCAAAAATTTGATGAAACAAATCCAAGGC**	4	PPR-A: +555 to +508
EFg24	**GCCTTGGATTTGTTTCATCAAATTTTTGAAACGACATGTAGACCAGATGTCCTAACGTTC**	4	PPR-A: +508 to + 555
Rg24Ecostop	**CTCTGAATTCGAGTGCAGCTTTCTTTTTTTTAACC**	4	PPR-A: +2456 to +2432
g24swapA	**GTCAGCATATTGCGGATAGTAAT**	5	PPR-A: +1970 to +1948
g2624FswapB	**ATTACCATCCGCAATATGCTGAC**	5	PPR-A: +1948 to +1970
P3g24g26Reverse	**ACCCTCGCGGCAAAGACC**	6	Rfo: +594 to + 577
P3g2426swapF	**GGTCTTTGCCGCGAGGGT**	6	Rfo: +577 to +594
g26RUTRA	**GAAGAAAATCTTTGATCATTCAGGTATCAGGATACACCAC**	7	Rfo: +2210 to +2189**<br>+1953 to +1939
g26FUTRB	**TGAATGATCAAAGATTTTCTTC**	7	Rfo: +2189 to +2210
Apa1Sal1F02	GAGAGGGCCCGTCGACGCCAAATACAACCCGAACTAATGG	8	PPR-A: -1125 to -1102
g24RXho1F03	CTCTCTCGAGACTCGAATCTGCAAACCCTAGCCAACAT	8	PPR-A +22 - +1

*Location relative to the initiation codon in either *Rfo* or *PPR-A.* Sequences designated as extending from a smaller to a larger nucleotide number prime extension in the direction of the 3’ UTR; sequences designated as extending from a larger to a smaller nucleotide number prime in the direction of the promoter.

**Primers corresponding to two distinct but nearby sequences were used in the generation of deletion mutations (constructs 3 and 7)
