# Supplementary material for: In vivo functional analysis of a nuclear restorer PPR protein
Source: BMC Plant Biol. 2014 Nov 18;14:313. doi: 10.1186/s12870-014-0313-4 (PMC4240901; doi:10.1186/s12870-014-0313-4)
Supplement: Additional file 6: — Generation of genetic constructs. [file 12870_2014_313_MOESM6_ESM.pdf]

GENERATION OF GENETIC CONSTRUCTS

Genetic constructs were assembled through site-specific or domain/promoter swapping approaches involving successive PCR reactions, as outlined in references [37, 38]. Two initial PCR reactions generated products that corresponded to partially overlapping portions of the intended final construct. The third PCR reaction which generated a final joined product using the products of the initial reactions as templates, was performed with an increased extension time of 5 minutes per cycle. Amplification was accomplished using the high fidelity Pwo DNA polymerase (Roche Diagnostics, Laval QC, Canada) and products were cleaved with the restriction endonucleases cloned into either pZErO-2 or pRfoP and sequenced (McGill University and Genome Quebec Innovation Centre) prior to introduction into the plant transformation vector to ensure the accuracy of the final construct and to confirm that no errors were introduced during the amplification reactions. The strategy involved in generating amplicons directed mutations as exemplified by construct 3 is outlined in Figure S3. The overall cloning strategy is illustrated in Supplementary Figure S4.

1. wtRfo. The base construct for the development of mutant versions of *Rfo* consisted of a 5,387 bp fragment extending from a site 1479 bp upstream of the *Rfo* initiation codon (nucleotide -1479) to a site 274 bp downstream of the termination codon (nucleotide +2,465) and spanning the promoter, 5′ UTR, coding, 3′ UTR and polyadenylation sequences. This fragment was amplified from the *Rfo*-containing Bgl-5 construct that transgenically suppresses *ogu* CMS in *B. napus* [16], using as a forward primer <Sal1PF> and as reverse primer <BRg26Eco>; Sal1 and EcoR1 recognition sites, respectively, were incorporated near the 5′ ends of these primers (the

sequences of the different primers used to generate genetic constructs are given in Supplementary Table 2). The resulting products were digested with Sal1 and EcoR1 and introduced into the *B. napus* plant transformation vector pRD400 [39].

2. RfoP. To facilitate the expression of variant forms of Rfo, a utility clone (*RfoP*) consisting of the *Rfo* promoter and 5′ UTR region extending from the 5′ end of *wtRfo* to the nucleotide immediately upstream of the initiation codon was generated by amplification of the region using Pf as the forward primer and Pr as the reverse primer. The Pf primer had Apa1 and Sal1 sites, and the Pr primer an Xho1 site, incorporated into their 5′ ends. The resulting amplicon was cleaved with Apa1 and Xho1 and cloned into the corresponding sites in the bacterial transformation vector pZErO-2 (Invitrogen Life Technologies, Burlington, ON).

3. RfoΔ. This construct was intended to evaluate the effect of introducing the 12 bp deletion found in the domain 4 coding sequence of PPR-A and the *rfo* allele into the *Rfo* coding sequence. Two independent PCR reactions were performed to generate products corresponding to the N-terminal and C-terminal coding regions of the mutant form, which were then joined by a third PCR reaction. Other mutant forms of Rfo were made using a similar strategy. The region of *Rfo* extending from the initiation codon to codon 187 was first amplified (PCR1) using primers <Fg26Xhostart> and <reverseg26> (PCR1); the first primer incorporated an Xho1 site at its 5′ end and the second primer corresponds to the inverse complement of nucleotides encoding amino acids 167 through 187, but with a deletion encompassing nucleotides encoding amino acids 177 through 180. A second PCR reaction (PCR2) was performed with <Bforwardg26>, a primer that is the inverse complement of <reverseprimerg26>, and <BRg26Eco> to amplify the

remainder of the gene including the 3′ UTR and polyadenylation sequence. The products of the two reactions were purified using a PCR clean-up kit (Qiagen, Toronto ON) and 20ng of the PCR1 product was mixed with 60ng of the PCR2 product to serve as a template for PCR 3. PCR3 was performed using primers <Fg26Xhostart> and <BRg26Eco> in conjunction with an oligonucleotide corresponding to the 3' end of the PCR1 product and extending across the 5′ end of the PCR2 product to amplify an ~2.4 kb product that extended from the *Rfo* initiation codon to the polyA sequence, with an Xho1 site at one end at an EcoR1 site at the other.

4. RfoP::PPR-A(1-3)::Rfo(4)::PPR-A(5-18–3'UTR). This construct was designed to determine the effect of introducing into the *PPR-A* gene the *Rfo* sequence encoding the four amino acids that are missing in its domain 4 on its capacity to restore fertility. PCR1 was performed on the cloned g24 sequence [16] spanning the *PPR-A* gene using a forward primer that allowed downstream extension from the initiation codon <DFg24Xhostart> with a reverse primer encompassing the additional 12 bp found in the Rfo domain 4<DRg24>; this product extended from the PPR-A initiation codon to codon 185 and contained an Xho1 site upstream of the initiation codon. PCR2 employed a forward mutant primer <EFg24> complementary to <DRg24> and the reverse primer <g24EcoR1stop>; this product extended from codon 170 of PPR-A to a site 264 nucleotides downstream of the termination codon and included the 3′ UTR, the poly A site and an EcoR1 codon. Finally, PCR 3 was performed using as templates the products of PCR1 and PCR2 with <DFg24Xhostart> and <Rg24Ecostop> as primers to produce the final product

5. *RfoP::PPR-A(1-3)::Rfo(4)::PPR-A(5-17)::Rfo(18-3'UTR).* This construct was designed to determine the effect of replacing the terminal PPR domain coding and 3′ untranslated regions of construct 4, above, with the corresponding portions of Rfo. Using construct 4 as template, PCR1 employed primers <DFg24Xhostart> and <g24swapA> to generate a product extending from the PPR-A initiation codon through PPR-A codon 655, with additional nucleotides corresponding to Rfo codons 652 through 657 and an Xho1 site adjoining the initiation codon. PCR2 employed the *wtRfo* construct as template with primers <g2624FswapB> and <BRg26Eco> to generate a product that overlaps with the downstream 18 nucleotides of the PCR1 product and extends to cover the Rfo the 3′ UTR and polyA site. PCR3 employed the products PCR1 and PCR2 as template with primers <DFg24Xhostart> and <BRg26Eco> to generate the final product.

6. *RfoP::Rfo (1-4)::PPR-A(5-17)::Rfo(18-3'UTR).* This construct was designed to determine the effect of replacing the first 3 PPR domains derived from PPR-A in construct 5 with the corresponding domains of *wtRfo*. The *wtRfo* construct was used as a template for PCR1 to generate a product extending from the initiation codon to codon 198, using the forward primer <Fg26Xhostart> and the reverse primer <P3g24g26Reverse>. PCR2 generated an overlapping product extending to the polyA site of construct 5 with the forward primer <P3g2426swapF>, which is complementary to <P3g24g26Reverse>, and the reverse primer <BRg26Eco>. PCR3 employed the products of PCR1 and PCR2 as template with primers < Fg26Xhostart> and <BRg26Eco> to generate the final product.

7. *RfoP::Rfo(1-17)::Rfo(UTR).* This construct was designed to test the effect of deleting only the C-terminal PPR domain on Rfo activity. PCR1 employed the forward primer <Fg26Xhostart>

with a reverse primer <g26RUTRA> and the wtRfo construct as template. Nucleotides 1 through 22 of the reverse primer are the inverse complement of the sequence extending from and including the Rfo termination codon to a site 19 nt downstream, and nucleotides 23 through 37 are the inverse complement of the sequence spanning codons 648 through 651. PCR2 employed the forward primer <g26FUTRB> which is complementary to nucleotides 1 – 22 of <g26RUTRA> together with the reverse primer <BRg26Eco>. PCR3 used the products of PCR1 and PCR2 as template together with primers <Fg26Xhostart > and <BRg26Eco> to produce a product that joined the region extending from the initiation codon through codon 651 to the region extending from the termination codon across the 3′ UTR and polyA sequence. The sequence contained a single continuous open frame encoding amino acids 1 through 651 of Rfo.

8. PPR-AP::Rfo (1-18, 3'UTR). This construct was designed to test the effect of using the PPR-A promoter to express the wtRfo protein. The region of the *wtRfo* construct extending from the initiation codon through the 3′ UTR/poly A sequence was amplified using primers <Fg26Xhostart > and <BRg26Eco> and the product of this reaction was digested with Xho1 and EcoR1 (New England Biolabs, Whitby ON) and cloned into the corresponding sites in pZErO-2. A region of the PPR-A region extending from a site 1,125 nucleotides upstream of, to a site just downstream of, the PPR-A initiation codon was amplified using the forward primer <Apa1Sal1F02>, which had Apa1 and Sal1 sites at its 5′ terminus, and the reverse primer < g24RXho1F03>, which had an Xho1 site at its 5′ terminus. The product was digested with Xho1 and Apa1 (New England Biolabs) and cloned into the pZErO-2 clone containing the wtRfo sequence.
